# Ethical and Legal Considerations in the Refusal of Antipsychotic Treatment During Pregnancy: A Case Report

**DOI:** 10.7759/cureus.114597

**Published:** 2026-08-16

**Authors:** Alexander Tam, Macey Hughes, Derry Brinley

**Affiliations:** 1 Medicine, Noorda College of Osteopathic Medicine, Provo, USA; 2 Psychiatry, Universal Health Services (United States), Orem, USA

**Keywords:** antipsychotics in pregnancy, decision-making capacity, forced medication, maternal-fetal ethics, perinatal psychiatry, psychiatric emergency in pregnancy, treatment refusal

## Abstract

Acute psychosis during pregnancy creates a complex clinical scenario that requires simultaneous consideration of maternal psychiatric stabilization and fetal well-being. Guidance remains limited on the management of acutely psychotic pregnant patients who lack decision-making capacity and refuse care, particularly in settings without on-site obstetric resources. We describe the case of a 35-year-old woman with schizoaffective disorder and post-traumatic stress disorder who was involuntarily committed for acute behavioral disturbance and found to be approximately 14 weeks pregnant during evaluation. She remained acutely psychotic, with persistent paranoid and grandiose delusions, impaired self-care, and disruptive, sometimes aggressive behavior. A formal capacity assessment determined that she lacked the capacity to make informed decisions about psychiatric treatment or pregnancy-related care. She refused antipsychotic medication, laboratory testing, and prenatal care, and de-escalation efforts were unsuccessful. Because the treating facility lacked on-site obstetric services and pharmacologic treatment carried fetal risks that the patient could not weigh, the team pursued transfer to a facility with both psychiatric and obstetric capabilities rather than a forced-medication hearing. After 20 days, she was transferred under commitment to a state hospital. This case illustrates that pregnancy transforms an already difficult involuntary-treatment decision into a multidimensional legal, ethical, and systems problem and highlights that early pregnancy screening, predefined transfer pathways, and multidisciplinary review should guide management when acute psychosis and pregnancy intersect.

## Introduction

Perinatal mental health conditions are common and contribute significantly to maternal morbidity and mortality, prompting recommendations for routine screening and early identification during pregnancy and the postpartum period [1]. Severe psychiatric illness in this population is associated with impaired self-care, inadequate prenatal engagement, and an increased risk of harm to both the patient and fetus [2]. Clinical management requires balancing the risks of untreated psychiatric illness against the potential risks of pharmacologic treatment [3]. Although available data suggest that antipsychotic use during pregnancy does not meaningfully increase the risk of congenital malformations after adjustment for confounders [4], both medication exposure and untreated illness carry clinically significant risks, and the existing evidence base remains limited [5].

These challenges are amplified when a patient lacks decision-making capacity, as interventions aimed at maternal stabilization may have foreseeable implications for the fetus. Despite increasing attention to medication safety in pregnancy, guidance remains limited on the management of acutely psychotic pregnant patients who lack capacity and refuse care, particularly in settings without multidisciplinary resources. This case report centers on the intersection of legal, ethical, and perinatal psychiatric considerations. What happens to the standard framework for treatment over objection when the patient's incapacity extends to decisions that may affect a fetus she cannot yet meaningfully weigh? We present this case to examine how capacity, patient autonomy, and institutional responsibilities interact when psychiatric emergency and pregnancy coexist, and to consider what systems-level safeguards might better support this decision-making process.

## Case presentation

A 35-year-old woman with a history of schizophrenia (later revised to schizoaffective disorder during hospitalization) and post-traumatic stress disorder was brought to the emergency department by law enforcement for evaluation of acute behavioral disturbance after reportedly repeatedly stabbing her mattress with a knife. The toxicology screen obtained in the emergency department was negative, and no other significant medical comorbidities were identified. She was transferred from the emergency department to an inpatient psychiatric facility under an existing involuntary commitment order after the referring county mental health authority determined it could not safely manage her care. Given her existing court-ordered psychiatric commitment, medical decision-making authority was deferred to the treating facility rather than to the patient or family. Her next of kin, her mother, was aware of the admission; the patient did not have a spouse or partner involved in her care, and family involvement in decision-making was accordingly minimal.

On presentation, the patient exhibited disorganized behavior and impaired engagement, initially sitting with her back turned to the interviewer and her face covered. During the latter portion of the interview, she spoke aloud in alternating perspectives, frequently referring to herself in the third person. She declined to participate meaningfully in questioning and was unable to provide a coherent history. She expressed distrust of the “industrial medical complex” and had previously refused evaluation by obstetric services before transfer.

Pregnancy was confirmed on ultrasound, with an estimated gestational age of approximately 14 weeks; the patient had previously provided inconsistent reports of her gestational age. This finding significantly altered the clinical context. The treating facility had no on-site obstetric services or immediate access to obstetric consultation, while the patient remained acutely psychotic, with persistent paranoid delusions that other patients were stealing her belongings, that her food was being tampered with, and that nursing staff were manipulating and resealing her medications. Additional delusional content included grandiose, religious, and sexual themes. Despite the absence of on-site obstetric services, pregnancy-related care available within the facility included prenatal treatment, maternal monitoring, and medication review with consideration of pregnancy.

Her behavior was frequently disruptive and at times physically aggressive, including throwing objects at staff. She demonstrated impaired self-care and judgment, intermittently refusing to eat because she believed the food provided was beneath her status. She was frequently verbally abusive, performed rituals such as pouring salt on her roommate's bed, and loudly responded to internal stimuli in various accents. During hospitalization, her clinical presentation was also notable for symptoms consistent with mania, including distractibility, impulsive behavior, grandiosity, flight of ideas, and pressured speech. In conjunction with her longitudinal psychiatric history, including a prior prolonged psychiatric hospitalization, the presence and temporal relationship of prominent mood symptoms and psychosis supported revision of her diagnosis from schizophrenia to schizoaffective disorder.

A formal capacity assessment, guided by standard criteria for evaluating a patient's ability to understand relevant information, appreciate its consequences, reason about treatment options, and communicate a consistent choice [6], found that the patient lacked the capacity to make informed medical decisions. She was specifically unable to meaningfully engage in discussion of psychiatric treatment and pregnancy-related care. The patient refused antipsychotic medication, laboratory testing, certain aspects of monitoring, and prenatal care [7]; attempts at verbal de-escalation and other supportive measures (including providing a structured environment, group therapy, music therapy, and interventions to promote sleep) were unsuccessful. Clinicians were then faced with the question of whether to pursue a forced-medication hearing to administer treatment involuntarily. The presence of pregnancy introduced additional complexity, as pharmacologic treatment carried potential fetal implications, and the patient was unable to participate in decisions about those risks.

Given the absence of obstetric consultation, concerns about safely managing a high-risk pregnancy, and the ethical and legal complexity of initiating involuntary treatment in this context, the treatment team elected to pursue transfer to a facility with both psychiatric and obstetric capabilities. This decision was driven by the need for multidisciplinary evaluation, access to obstetric expertise, and a setting better equipped to address both maternal psychiatric stabilization and pregnancy-related care. After 20 days, the patient was transferred under commitment to a state hospital. At the time of transfer, she remained agitated, singing, yelling, and attempting to harm the EMS personnel transporting her.

## Discussion

Determination of capacity is among the first decisions to be made for any patient. This includes evaluating a patient's insight into her situation, logical reasoning, and ability to consent to treatment. Capacity should be assessed independently for each decision and reassessed throughout hospitalization, since psychiatric status may fluctuate with treatment and time. Loss of decision-making capacity does not automatically authorize every intervention a clinician believes to be beneficial. In psychiatric patients, as in any other patients, clinicians must still weigh the specific risks, benefits, and less restrictive alternatives for each proposed intervention. Pregnant patients with acute psychosis represent a unique and understudied population in whom severe psychiatric illness may result in loss of decision-making capacity, necessitating involuntary commitment and, in some circumstances, treatment over objection. In this case, the treatment decision extended beyond psychiatric stabilization, since administration of antipsychotic medication carried implications for fetal exposure and pregnancy management.

The specific nature of the fetal risks at stake merits closer examination, since it directly shapes the risk-benefit calculus in cases of treatment over objection. Current evidence does not support antipsychotics as teratogens. A 2026 systematic review and meta-analysis pooling 12 studies and more than 10 million pregnancies found no significant association between in utero antipsychotic exposure and congenital malformations [8]. The same analysis did identify a modest but statistically significant increase in the risk of preterm birth associated with antipsychotic exposure (OR: 1.35; 95% CI: 1.13-1.62) [8]. Taken together, these findings indicate that the fetal risks attributable to antipsychotic exposure are modest and primarily associated with preterm delivery rather than teratogenicity. This distinction is clinically important: it recalibrates what is being weighed against the well-documented maternal and fetal risks of prolonged untreated psychosis when treatment over objection is under consideration.

The central legal question is not simply whether involuntary medication is ever permissible, but whether its justification changes when treatment foreseeably affects both maternal psychiatric status and fetal exposure in a patient unable to articulate her pregnancy-related preferences. Current legal precedent supports involuntary medication in dangerous or incapacitated patients: Washington v. Harper establishes that involuntary psychiatric treatment may be justified when significant safety concerns or impaired decision-making capacity are present, subject to appropriate procedural safeguards [9]. Outside of immediate emergencies, however, judicial precedent places greater emphasis on protecting patient autonomy and procedural safeguards before treatment over objection is authorized [10]. These decisions establish important principles regarding involuntary treatment but offer limited guidance for situations in which the patient is pregnant, and treatment decisions carry obstetric implications. The specific legal calculus may also vary by jurisdiction and gestational age; however, our core recommendations regarding early screening and timely multidisciplinary involvement are not contingent on this variation.

The clinician must balance respect for patient autonomy against the ethical obligations of beneficence and nonmaleficence [11,12]. Importantly, untreated psychosis is not a neutral option: withholding treatment also carries significant maternal and fetal risk. Antipsychotics cross the placenta, and clinicians must weigh the potential fetal risks of medication exposure against the well-established risks of untreated severe psychiatric illness [13,14]. Effective treatment of severe psychosis may reduce the risk of self-harm, malnutrition, impaired prenatal care, and medical neglect, thereby improving maternal health and potentially fetal outcomes [15]. Less restrictive interventions, including verbal de-escalation, environmental modification, close observation, and specialist consultation, should be attempted whenever clinically appropriate before treatment over objection is pursued [16]. When available, family or partner involvement in decision-making should also be sought, both to support the patient and to provide collateral history that may inform capacity assessment and treatment planning.

Routine pregnancy screening should be considered during the admission process for reproductive-age patients presenting with acute psychosis [17]. In this case, pregnancy was discovered only after admission to our ward, and several weeks were required to secure placement at a facility with obstetric services. Placement at a psychiatric facility without obstetric resources limited access to multidisciplinary pregnancy care and complicated subsequent management; transfer to a facility with obstetric capabilities ultimately allowed maternal psychiatric care to proceed while minimizing foreseeable fetal risk through multidisciplinary evaluation. This case suggests that early pregnancy identification and predefined transfer pathways should be established when psychiatric facilities lack obstetric resources. When a pregnant patient requiring involuntary psychiatric hospitalization is admitted to such a facility, early transfer and prompt consultation with obstetrics or maternal-fetal medicine should be prioritized to facilitate coordinated multidisciplinary care. This report focuses specifically on recognition and pre-transfer decision-making, rather than on management following arrival at a higher level of care, which was outside our institution's direct observation and beyond this report's scope.

## Conclusions

Pregnancy transforms an already difficult involuntary-treatment case into a multidimensional legal, ethical, and systems problem. Loss of capacity does not eliminate the need for careful reasoning about maternal preferences, fetal impact, and least-restrictive alternatives. We recommend that psychiatric facilities without on-site obstetric services adopt the following practices: (1) universal pregnancy screening at admission for reproductive-age patients presenting with acute psychiatric illness; (2) early obstetric specialist consultation once pregnancy is identified; (3) ethical and legal review involving the treatment team and family, when available, before pursuing treatment over objection; and (4) transfer to a facility capable of providing both obstetric and psychiatric care when the treating facility cannot meet both needs. These protocols, combined with multidisciplinary review rather than reflexive coercion or reflexive deferral, should guide management when acute psychosis and pregnancy intersect.
